# Unforeseen sequela of a traditional remedy: A garlic burn case report

**DOI:** 10.51866/cr.802

**Published:** 2025-05-10

**Authors:** Shamsudin Nor Amal Lina, Chee Hon Yee, Yoon Chin Yap

**Affiliations:** 1 MD, MEmMed, Emergency Department, Queen Elizabeth Hospital, Ministry of Health Malaysia, Jalan Penampang, Kota Kinabalu, Sabah, Malaysia. E-mail: angelineyap7@gmail.com; 2 MBBS, Emergency Department, Queen Elizabeth Hospital, Ministry of Health Malaysia, Jalan Penampang, Kota Kinabalu,Sabah, Malaysia.; 3 MD, Emergency Department, Queen Elizabeth Hospital, Ministry of Health Malaysia, Jalan Penampang, Kota Kinabalu, Sabah, Malaysia.

**Keywords:** Garlic, Burn, Natural remedy

## Abstract

Garlic is one of the most popular natural home remedies for a variety of illnesses. Many different cultures have recognised its beneficial properties as a herbal remedy since ancient times. Nevertheless, inappropriate usage of garlic can cause unwanted side effects despite its numerous positive qualities. Although they may appear to be the least dangerous, natural remedies like garlic can also cause burn themselves. Garlic typically resulted in second-degree burns, but in some cases, necrotic tissue could also develop. This case report described burn injuries caused by the application of raw garlic. We aimed to demonstrate the possible adverse effects of using garlic as a natural home remedy as well as to educate the public and healthcare providers about inappropriate usage of natural home remedy for self-treatment.

## Introduction

Herbal medicine is often utilised as an alternative or complementary treatment especially in Asian cultures despite the advancement of modern medicine. Garlic *(Allium sativum* L. Fam. Liliaceae) is one of the most commonly used herbs and has been used as a traditional remedy for various ailments due to its perceived medicinal properties. However, it is associated with various adverse effects, which include gastrointestina^l^ symptoms, hypocoagulation and cutaneous reactions.^1^ Herein, we report the case of a 3-month-old child who sustained an unintentional chemical burn after raw garlic was applied topically as a home remedy for cough.

## Case presentation

A 3-month-old boy was brought to our emergency department by his mother with a chief complaint of multiple blisters and swelling developing on the child’s feet. The child was otherwise well clinically and did not have any fever prior to presentation. He had cough for the past 2 weeks, and the mother sought to apply garlic topically on the boy’s feet to complement symptomatic treatment given by his general practitioner, as she was worried about the prolonged cough. The patient’s mother used a home remedy by applying one clove of freshly crushed raw garlic to the boy’s feet and covering it with a band-aid overnight for 6 hours prior to presentation. The mother noticed that her child’s feet became red, with new vesicles and blisters appearing at the site previously covered by the band-aid. The child was immediately brought to the emergency department for further treatment when the mother discovered these skin changes on the boy.

**Figure 1:**
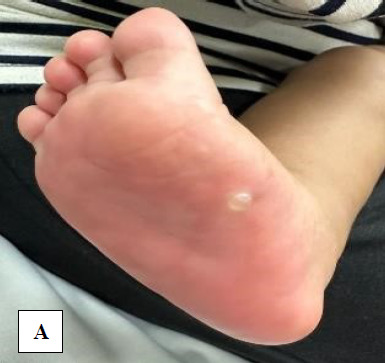

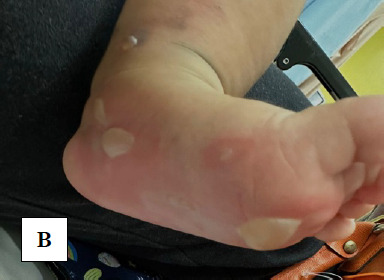

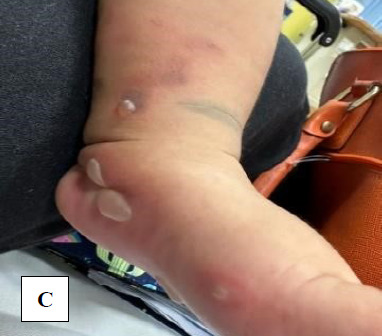
Bullae on the right foot (A) and left foot (B and C) upon presentation to our department.

Upon examination of the child, both of his feet were erythematous and warm in addition to the presence of multiple blisters over his bilateral soles, with the largest blister measuring 3x3 cm over the left foot (Figure 1B and 1C) and 1x1 cm over the right foot (Figure 1A). The patient was diagnosed with irritant contact dermatitis, also commonly known as ‘garlic burn’. Wound irrigation was subsequently carried out, and the patient was discharged with instructions to return for daily dressing with normal saline at the nearest clinic. The patient’s mother was also advised to stop with the application of garlic or any other herbal medication on her son. The patient’s wound eventually improved over the course of the next few days.

## Discussion

A burn can occur due to thermal, chemical, electrical or radiation injury.^2^ Scalding is the most prevalent type of burn injury in the Malaysian population, followed by contact and flame injuries. Howecer, only a small percenlage of cases are reported as chemical burns,^3^ and *a* mere 4% of these cases are attributed to the use of herbal medication. Many studies have reported life-threatening chemical burns when traditional remedies are utilised.^4^ A study conducted in a Korean population showed that traditional remedies such as moxibustion, glacial acetic acid, garlic substances and herbal extracts *(Pulsatilla koreana*) could lead to burn injuries.^5^

People who live in socioculturally and economically underdeveloped regions tend to seek alternative or traditional treatment.^6^ Historically, garlic has been utilised in traditional medicine, as it is believed to have antiviral, antibacterial and antifungal effects. Recent studies have discovered that garlic has anticancerous, immunomodulatory and antioxidant properties in addition to being able to treat hypertension, lower cholesterol/lipid levels and reduce the risk of coronary events at the age of 50 years.^7,8^

Despite its numerous benefits, garlic is associated with undesirable side effects such as nausea, heartburn, diarrhoea, asthma, tachycardia and insomnia. Topical exposure to raw garlic may result in the development of contact dermatitis, skin blisters and ulceronecrotic lesions as well as chemical burns if directly applied to the skin and mucosa.^9-11^ Most reported garlic burn cases are second-degree partial-thickness burns, as in our patient. Erythema and vesicobullous changes are noted in the majority of cases, while prolonged use of garlic in young children might result in necrotic ulcers.^12^

Most garlic burn cases have not used additional ingredients except raw garlic.^11,13^ Intact garlic cloves consist of alliin and the enzyme alliinase, which are stable in dry and undamaged conditions. However, mechanical damage such as chopping, crushing, incising and grounding renders the chemical compounds unstable, especially when exposed to heat, as the enzyme alliinase converts alliin into allicin.

The extent of garlic tissue destruction correlates with the amount of allicin formeU, but the amount of reactive allicin is impossible to quantify and will vary in each instance. Disulfide bonds are formed between the sulphur group of allicin and thiol groups of enzymes and other structures, causing the effect of garlic burns. Therefore, the main culprit for chemical garlic burns is these volatile organic sulphur compounds includin^u^ Oiallyl disulfide, which are Oormed aOtet mechanical damage. Moreoaer, the common denominator in most reported cases is the usage of fresh garlic cloves, which have been mechanically damaged in alignment with the described chemistry. The effect of sulphur compounds can also be further enhanced by allicin and other compounds reacting rapidly with macromolecules when patients apply garlic topically and fix it in place.^12^ This was seen in our patient, as freshly crushed garlic was applied to his feet and fixed in place with the use of a band-aid, resulting in chemical burns.

Garlic burns can happen across different age groups. The systematic review by Hitl et al. examined the demographics of patients who sustained garlic burns and found a wide range of age distribution, with patients ranging from a 3-month-old infant to an 80-year-old woman.^12^ Furthermore, the reasons for garlic application varied from therapeutic purposes to intentional child abuse, particularly in younger children or babies.^5,12,14^ Therefore, good history-taking is crucial to exclude the possibility of nonincidental injury in garlic burns.

The severity of garlic skin burns is determined by some factors such as the amount and freshness of garlic, duration of exposure, application of occlusive dressing, the presence of pre-existing skin conditions and sensitivity of the skin. In this case, the severity of the burn likely worsened due to the usage of occlusive dressing on top of the delicate nature of the child’s skin. The average time required for burn development is 6-8 hours in children and longer in adults. This is congruent with our patient, a 3-month-old boy who developed chemical burn 6 hours after garlic was applied topically to his feet. Furthermore, prolonged topical exposure to garlic (over 2 days or more) can result in burns with necrosis.^11,12^

Garlic burns can be treated with daily dressing or application of anti-inflammatory agents such as topical corticosteroids. Oral antibiotics can be prescribed if secondary bacterial infection has developed or is suspected. Symptoms usually resolve within 2 weeks.^15^

## Conclusion

This case report emphasises the potential risks associated with natural remedies containing garlic when used topically. Healthcare providers should keep in mind that garlic can be a potential causative agent for chemical burns especially with a positive history of topical garlic application to ensure accurate diagnosis and treatment. Furthermore, it is important to educate the public about the possible adverse effects of herbal medicine if used inappropriately.
